# Supplementary material for: Reconciling Mining with the Conservation of Cave Biodiversity: A Quantitative Baseline to Help Establish Conservation Priorities
Source: PLoS One. 2016 Dec 20;11(12):e0168348. doi: 10.1371/journal.pone.0168348 (PMC5173368; doi:10.1371/journal.pone.0168348)
Supplement: S1 Dataset — (ZIP) [file pone.0168348.s002.zip › Taxa/Serra Norte/SN_2013/N4WS/Lista N4WS-50.pdf]

CAVIDADE N4WS-0050/0051

Classe	Ordem	Fam/Outros	Gên/Outros	Espécie	S-U	U-S	Zon.
Arachnida	Acari	(Oribatida)		sp.2	X		E
Arachnida	Acari	Rhagidiidae		sp.1		X	E
Arachnida	Araneae	Araneidae	*Alpaida*	*septemmammata*	X	X	E/P
Arachnida	Araneae	Araneidae	*Alpaida*	*smila*	X		E
Arachnida	Araneae	Corinnidae		jovem	2		E
Arachnida	Araneae	Ctenidae	*Ctenus*	sp.1	1	2	E
Arachnida	Araneae	Dipluridae		jovem		1	E
Arachnida	Araneae	Filistatidae		sp.1	X	X	E/P
Arachnida	Araneae	Mysmenidae	*Microdipoena*	sp.1		X	E
Arachnida	Araneae	Pholcidae	*Mesabolivar*	*aurantiacus*		X	E
Arachnida	Araneae	Pholcidae	*Mesabolivar*	*eberhard*	X		E
Arachnida	Araneae	Pholcidae	Ninetinae	sp.1		X	E
Arachnida	Araneae	Prodidomidae		jovem		X	E
Arachnida	Araneae	Scytodidae	*Scytodes*	*eleonorae*		1	E
Arachnida	Araneae	Scytodidae	*Scytodes*	*globula*	20	17	E/P
Arachnida	Araneae	Scytodidae	*Scytodes*	sp.3		1	E
Arachnida	Araneae	Theraphosidae		jovem		7	E
Arachnida	Araneae	Theridiidae	*Achaearanea*	sp.1	X	X	E/P
Arachnida	Opiliones	Escadabiidae		sp.1		X	E
Arachnida	Opiliones	Stygnidae	*Protimesius*	*laevis*	1		E
Arachnida	Pseudoscorpiones	Bochicidae		sp.1	X		E
Arachnida	Pseudoscorpiones	Chthoniidae	*Pseudochthonius*	sp.1		X	E
Arachnida	Pseudoscorpiones	Olpiidae		sp.1		X	E
Entognatha	Collembola	Entomobryidae		sp.1		X	E
Entognatha	Collembola	Entomobryidae		sp.6		X	E
Entognatha	Collembola	Isotomidae		sp.1		X	E
Entognatha	Collembola	Paronellidae		sp.1	X	X	E
Entognatha	Collembola	Paronellidae		sp.3	X		E
Entognatha	Collembola	Paronellidae		sp.4		X	E
Entognatha	Diplura	Campodeidae		sp.1	X		E
Insecta	Blattodea			jovem		1	E
Insecta	Coleoptera	Carabidae		sp.13	X		E
Insecta	Coleoptera	Hydrophilidae	Sphaeridiinae	sp.13		X	P
Insecta	Coleoptera	Tenebrionidae		jovem		X	E
Insecta	Diptera	Cecidomyiidae	Cecidomyiinae	sp.	X	X	E
Insecta	Diptera	Ceratopogonidae		sp.	X	X	E/P
Insecta	Diptera	Culicidae	*Culex*	sp.		X	E
Insecta	Diptera	Dolichopodidae		sp.	X		E
Insecta	Diptera	Drosophilidae	*Drosophila*	*eleonorae*	X	X	E/P
Insecta	Diptera	Ephydridae		sp.	X		E
Insecta	Diptera	Milichiidae		sp.	X		P
Insecta	Diptera	Muscidae		jovem	X		P
Insecta	Diptera	Phoridae	Metopininae	sp.	X		E
Insecta	Diptera	Psychodidae	*Telmatoscopus*	sp.	X		E/P
Insecta	Diptera	Therevidae		sp.	X		E
Insecta	Diptera	Tipulidae	Tipulinae	sp.	X		E
Insecta	Heteroptera	Cydnidae	*Pangaeus*	sp.1		X	E
Insecta	Heteroptera	Reduviidae	Emesinae	jovem		1	E
Insecta	Heteroptera	Reduviidae	Reduviinae	jovem		3	E

Insecta	Heteroptera	Veliidae	*Paravelia*	sp.1		X	E
Insecta	Hymenoptera	Chalcidoidea		sp.1	X		E
Insecta	Hymenoptera	Chalcidoidea		sp.2		X	E
Insecta	Hymenoptera	Formicidae	*Apterostigma*	sp.1	X		E
Insecta	Hymenoptera	Formicidae	*Brachymyrmex*	sp.1	X		E
Insecta	Hymenoptera	Formicidae	*Dolichoderus*	aff. *bispinosus*	X	X	E/P
Insecta	Hymenoptera	Formicidae	*Gnamptogenys*	*striatula*		X	E
Insecta	Hymenoptera	Formicidae	*Pachycondyla*	*striata*	1	3	E/P
Insecta	Hymenoptera	Formicidae	*Pheidole*	sp.2	X		E
Insecta	Hymenoptera	Formicidae	*Wasmannia*	sp.1		X	E
Insecta	Isoptera	Termitidae	*Nasutitermes*	sp.	X		E
Insecta	Lepidoptera	Gelechioidea		sp.2		X	E
Insecta	Lepidoptera	Noctuoidea		sp.1	3		E
Insecta	Lepidoptera	Tineoidea		sp.3		X	E
Insecta	Neuroptera	Myrmeleontidae		jovem		3	E
Insecta	Orthoptera	Phalangopsidae	*Paraclodes*	sp.	3		E
Insecta	Orthoptera	Phalangopsidae	*Phalangopsis*	sp.	100	100	P
Insecta	Psocoptera	Psocomorpha		jovem		X	E
Insecta	Psocoptera	Psyllipsocidae	*Psyllipsocus*	sp.1		X	E
Insecta	Thysanura	Nicoletiidae		sp.1		X	E
Malacostraca	Isopoda	Dubioniscidae		sp.1		X	E
Malacostraca	Isopoda	Philosciidae		sp.2		X	E
Amphibia	Anura	Leptodactylidae	*Leptodactylus*	cf. *vastus*		1	E
Amphibia	Anura	Strabomantidae	*Pristimantis*	cf. *fenestratus*	8		E/P
Mammalia	Chiroptera	Emballonuridae	*Peropteryx*	sp.		3	P
Mammalia	Chiroptera	Furipteridae	*Furipterus*	*horrens*	10	5	P
Mammalia	Chiroptera	Phyllostomidae	*Carollia*	*perspicillata*	10	15	P
Reptilia	Squamata	Gekkonidae	*Thecadactylus*	sp.	1		E
